# Exploiting the potential of autophagy in cisplatin therapy: A new strategy to overcome resistance

**DOI:** 10.18632/oncotarget.3902

**Published:** 2015-05-06

**Authors:** Jesús García-Cano, Gorbatchev Ambroise, Raquel Pascual-Serra, Maria Carmen Carrión, Leticia Serrano-Oviedo, Marta Ortega-Muelas, Francisco J. Cimas, Sebastià Sabater, María José Ruiz-Hidalgo, Isabel Sanchez Perez, Antonio Mas, Félix A. Jalón, Aimé Vazquez, Ricardo Sánchez-Prieto

**Affiliations:** ^1^ Unidad de Medicina Molecular, Centro Regional de Investigaciones Biomédicas. Universidad de Castilla-La Mancha, Albacete, Spain; ^2^ INSERM U.1197/Université Paris-Sud/Equipe Labellisée Ligue Nationale Contre le Cancer, Hôpital Paul Brousse, Villejuif, France; ^3^ Departamento de Química Inorgánica, Orgánica y Bioquímica, UCLM. Facultad de Ciencias y Tecnologías Químicas-IRICA, Ciudad Real, Spain; ^4^ Fundación Parque Científico y Tecnológico de Castilla-La Mancha, Albacete, Spain; ^5^ Radiation Oncology Department, Complejo Hospitalario Universitario Albacete (CHUA), Spain; ^6^ Departamento de Química Orgánica, Inorgánica y Bioquímica, Facultad de Medicina, Albacete, Spain; ^7^ Unidad asociada de Biomedicina, UCLM-CSIC, Albacete, Spain; ^8^ Department of Biochemistry, School of Medicine, UAM/Biomedical Research Institute of Madrid, Madrid CSIC/UAM, Madrid, Spain; ^9^ Facultad de Farmacia, Universidad de Castilla-La Mancha, Albacete, Spain

**Keywords:** cisplatin, apoptosis, autophagy, synthetic lethality, monoplatin

## Abstract

Resistance to cisplatin is a major challenge in the current cancer therapy. In order to explore new therapeutic strategies to cisplatin resistance, we evaluated, in a model of lung cancer (H1299 and H460 cell lines), the nature of the pathways leading to cell death. We observed that H1299 displayed a natural resistance to cisplatin due to an inability to trigger an apoptotic response that correlates with the induction of autophagy. However, pharmacological and genetic approaches showed how autophagy was a mechanism associated to cell death rather than to resistance. Indeed, pro-autophagic stimuli such as mTOR or Akt inhibition mediate cell death in both cell lines to a similar extent. We next evaluated the response to a novel platinum compound, monoplatin, able to promote cell death in an exclusive autophagy-dependent manner. In this case, no differences were observed between both cell lines. Furthermore, in response to monoplatin, two molecular hallmarks of cisplatin response (p53 and MAPKs) were not implicated, indicating the ability of this pro-autophagic compound to overcome cisplatin resistance. In summary, our data highlight how induction of autophagy could be used in cisplatin resistant tumours and an alternative treatment for p53 mutated patient in a synthetic lethally approach.

## INTRODUCTION

Cisplatin (CDDP) is one of the most widely used drugs in cancer therapy. [1] Its mechanism of action has been deeply investigated, being the DNA molecules the main target of this drug. [2, 3] Since early 90's, it is known that apoptosis is the main mechanism by which CDDP exerts its cytotoxic effect [4] (For an excellent review, see ref. [5]). Nonetheless, resistance to this drug is still a major challenge in cancer therapy due to its wide use in the treatment of different types of tumours as well as its use in combination with other therapies. [1] Therefore, the search of new therapeutic alternatives to solve this problem will have an impact in the daily clinical practice. In this regard, novel molecules based on platinum are currently developed and, some of them, under clinical investigation like satraplatin, which is known to promote apoptosis to execute its therapeutic effect. [6] Furthermore, it has been proposed the use of novel targets for CDDP, like mitochondrial DNA, to overcome CDDP resistance rendering compounds like Platin-M which is able to promote an apoptotic response. [7]

Autophagy is the main mechanism for recycling cellular components. The most commonly studied type of autophagy is macroautophagy and there is already a vast knowledge about its molecular machinery (For a review, see ref. [8]). Recently, a growing body of evidences is implicating autophagy in cancer and its therapy. [9] There are several ways of how autophagy is related to cancer therapy. On the one hand, compounds able to trigger this biological response are considered novel and promising therapeutic approaches, being the mTOR inhibitors the best example (For a review, see ref. [10]). On the other hand, there are numerous examples of how autophagy is related to a resistant response as in the case 5-Fluorouracil, Doxorrubicin or ionizing radiation among others. [11–13] Even, in response to non DNA-damaging agents like Tyr-kinase inhibitors (vg. Sorafenib), it has been observed the induction of autophagy, [14, 15] indicating the broad implication of this process in cancer therapy. In this sense, it is noteworthy how compounds able to block autophagy are considered therapeutic agents, especially in combination with DNA damaging agents, as in the case of chloroquine, but with side effects that should be considered in future therapies. [16] In summary, the role of autophagy in cancer therapy seems to be dual, with implications either in sensitivity and in resistance depending on the system and the therapeutic agent used. [17, 18]

In this context, we decided to study the basis of CDDP resistance in an experimental model of lung cancer. Our data indicate that resistance to CDDP is due to the lack of a functional apoptotic response. However, this resistance correlates with the appearance of autophagy, which seems to be a mechanism of cell death poorly triggered by CDDP. Interestingly, in response of pro-autophagic compounds such as mTOR or Akt inhibitors, no differences were detected between cell lines. Indeed, a novel platinum-based compound monoplatin (MonoPt), which specifically promotes autophagy, was able to kill CDDP-resistant and sensitive cells in a similar fashion. This effect was independent of key players of the cellular response to CDDP such as p53 or MAPKs. Therefore, our results suggest that a synthetic lethality approach based on autophagy could open a new therapeutic window for tumours with a deficient response to apoptotic stimuli like CDDP.

## RESULTS

### Resistance to CDDP correlates with lack of apoptosis

**Figure 1:**
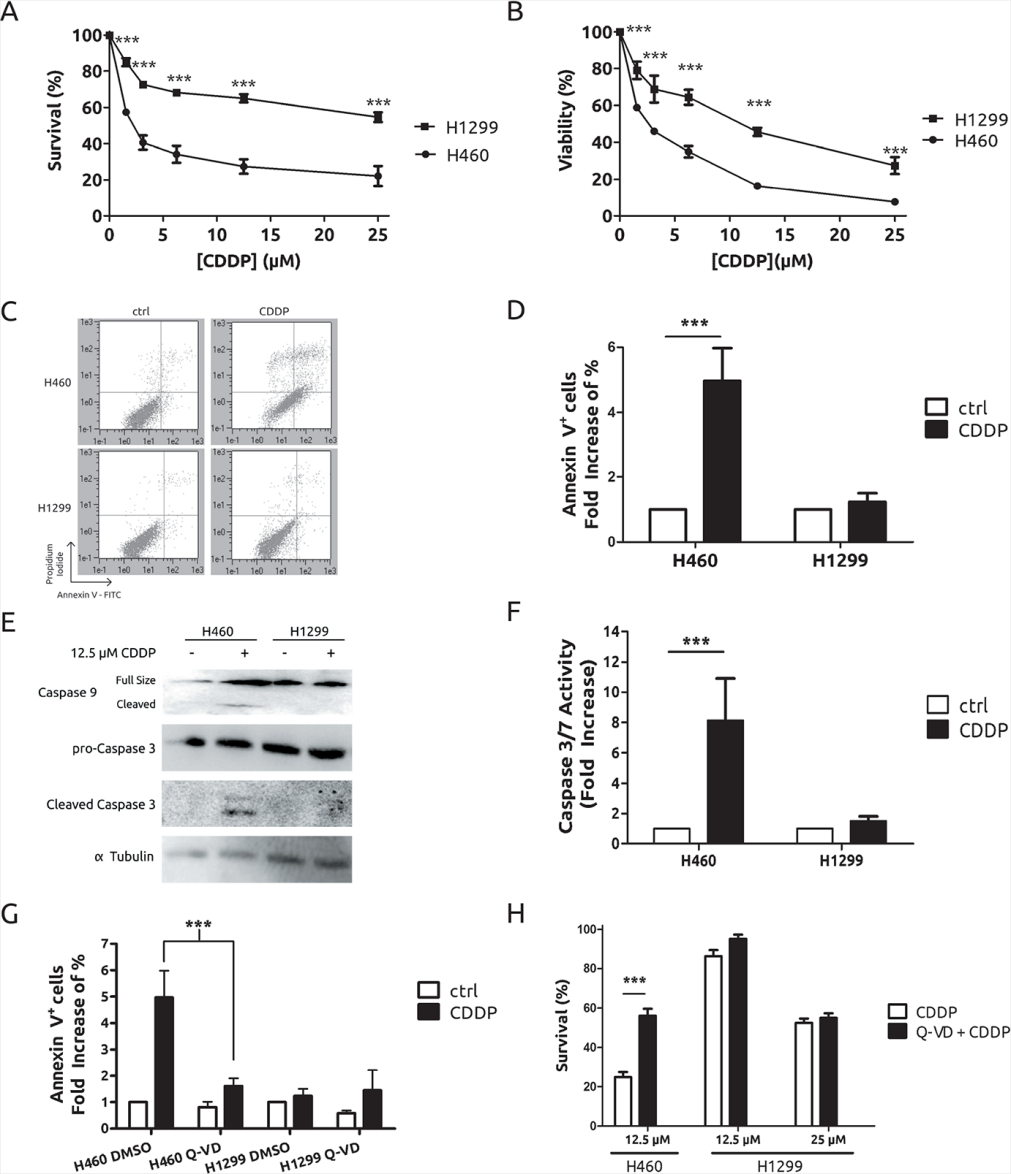
CDDP triggers cell death through the apoptotic pathway on H460 cells but not on H1299 cells Cells were treated for 48 h at the indicated concentrations and survival and viability were assessed by crystal violet **A.** or MTT **B.** respectively. **C.** Cells were treated with 12.5 μM CDDP for 36 h and stained with Annexin V-FITC/Propidium Iodide for cytometric assay. **D.** Results from 3 independent experiments conducted as in (C). **E.** Cells were treated with CDDP at indicated concentrations for 36 h and protein extracts were blotted with the indicated antibodies **F.** Cells were treated with 12.5 μM CDDP for 24 h and caspase 3/7 activity was evaluated. **G.** Cells were treated and processed as in (C) in the presence or absence of 10 μM Q-VD caspase inhibitor and plotted as in (D). **H.** Survival upon treatment with CDDP, at the indicated concentrations, in the presence or absence of 10 μM Q-VD caspase inhibitor measured by crystal violet 48 h after co-treatment.

To gain further insight into the basis of CDDP resistance in lung cancer we used H1299 and H460 cells. First, we performed a dose-response assay to CDDP in both cell lines. As expected, H1299 showed a great resistance by crystal violet method as well as by MTT (Figure 1A and 1B), in agreement with previous reports [19]. Considering that apoptosis is the main mechanism for cell death associated to CDDP we evaluated it by different methods in both experimental models. While H460 cells displayed a consistent induction of apoptosis by means of flow cytometry assays (Figure 1C and 1D), western blotting and caspase enzymatic activity (Figure 1E and 1F), no effect was observed in H1299 cells (Figures 1C, 1D, 1E, 1F). In the case of H460, no activation on caspase 8 was observed (data not shown) thus leading us to conclude that apoptosis was triggered through the extrinsic pathway. To fully prove our observations, we challenged a pan-caspase inhibitor like Q-VD-OPh (Q-VD). [20] Q-VD was able to promote a decrease in the apoptotic response in H460 cells that correlates with an increase in their viability. Interestingly, the same compound did not modify the response in H1299 (Figures 1G and 1H). Furthermore, another DNA-damaging agent such as ionizing radiation, known to promote apoptosis, was challenged in this experimental system showing again a correlation between resistance and lack of apoptosis (Supplementary Figure S1). In summary, this set of experiments demonstrate that the lack of apoptosis is a key mechanism in the resistance observed in H1299 cells.

**Figure 2:**
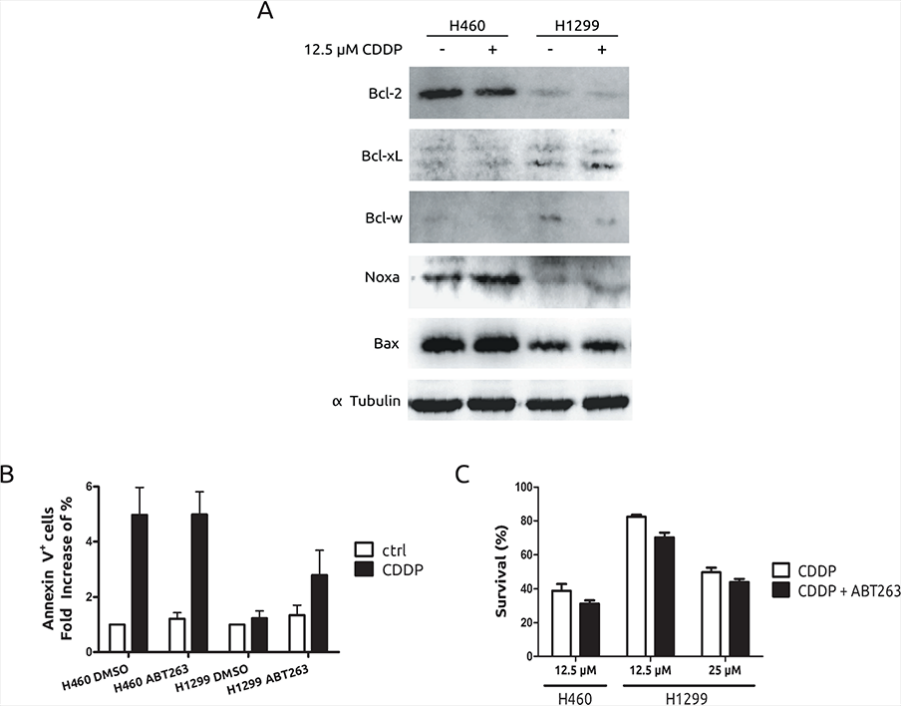
Pro- and anti- apoptotic protein expression pattern in H1299 and H460 cells **A.** Presence of Bcl-2 superfamily members and BH3-only proteins was evaluated by western blot after 36 h of CDDP treatment. **B.** Cells were treated with CDDP in the presence or absence of 20 nM of ABT263 and Annexin V staining was observed 36 h after by cytometry. **C.** Viability was evaluated in the same conditions as in (B) 48 h after by crystal violet method.

To fully understand CDDP resistance associated to H1299 cells we decided to analyse several anti- and pro-apoptotic proteins. As it is shown (Figure 2A), among the anti-apoptotic, Bcl-2 family proteins, Bcl-xL and Bcl-w showed a slight upregulation in H1299 cells while a marked downregulation was observed for Bcl-2. In the case of pro-apototic, BH3-only proteins, Noxa and Bax showed a marked downmodulation in H1299 cells, in agreement with a previous report that connects these proteins to CDDP response. [21] Indeed, the use of a BH3-mimetic compound such as ABT263, [22] showed no effect onto H460 cells, while in H1299, it was able to promote a discrete effect onto the apoptotic response with almost no effect on viability (Figures 2B and 2C). These last data suggest that a deregulated pattern in pro- and anti-apoptotic proteins could be a mechanism to partially explain the resistant phenotype observed in H1299 cells. However, it should coexist with other mechanisms to fully explain the acute resistance of these cells.

### H1299 cells undergo autophagy in response to CDDP

**Figure 3:**
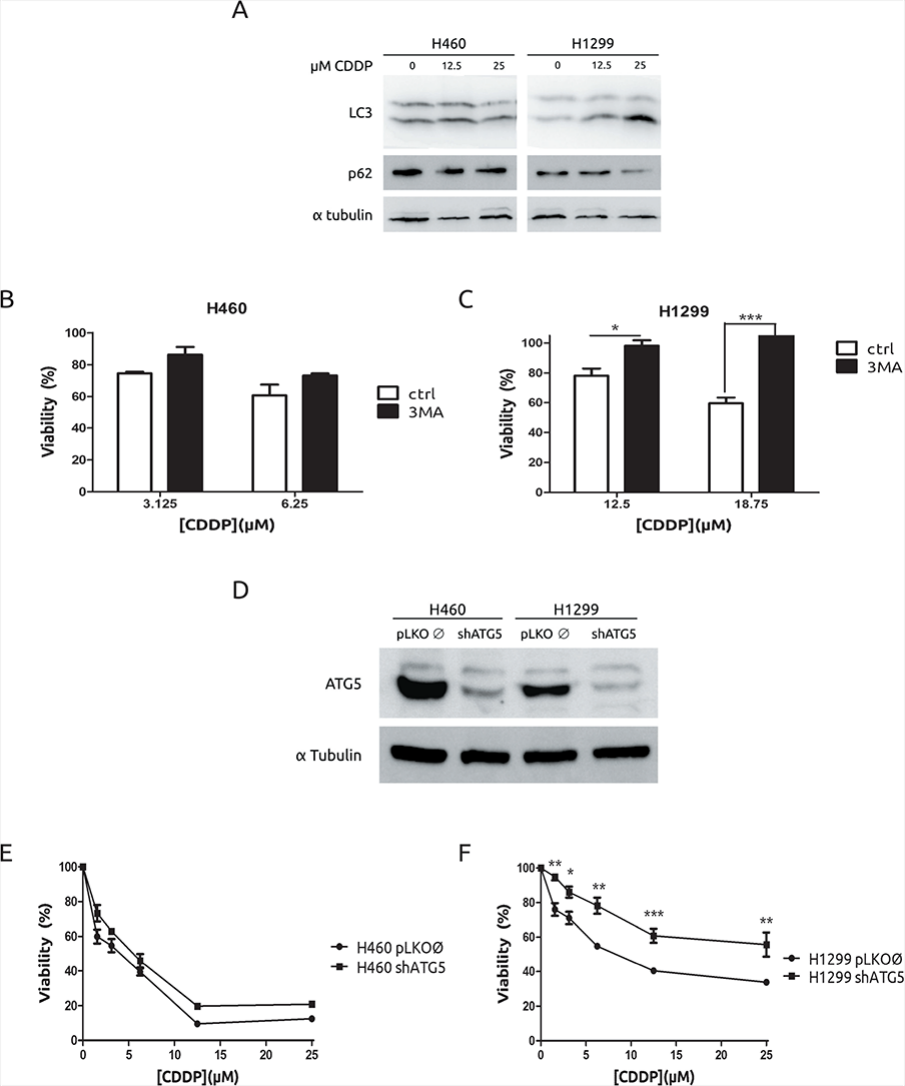
Autophagy is associated to cell-death in H1299 cells in response to CDDP **A.** H460 cells were treated for 36 h and protein extracts were blotted with the indicated antibodies. **B.** and **C.** Cells were treated with CDDP at the indicated concentrations in the presence or absence of 2.5 mM 3MA. Viability was assessed by MTT. **D.** Extracts from H460 cells and H1299 cells infected either with lentivirus carrying an empty vector or shRNA against Atg5 were blotted against ATG5. **E.** H460 cells and H1299 cells **F.** were infected as described in D and treated with the indicated concentrations of CDDP and viability was measured by MTT.

A growing body of evidences support that autophagy is a putative mechanism of resistance to CDDP. [23–25] Therefore, we decided to challenge this hypothesis in our experimental model by evaluating lipidation of LC3 and degradation of p62/SQTSM1 following standard procedures. [26] As it is shown, H1299 cells showed an increase in the lipidated form of LC3 (LC3-II) as well as a decrease of p62/SQSTM1 in response to CDDP, which were not observed in H460 cells (Figure 3A). In fact, transfection of GFP-LC3 in H1299 renders a pattern consistent with the induction of autophagy (Supplementary Figure 2A). To fully support the role of autophagy in CDDP resistance, H1299 and H460 cells were incubated in the presence of a known autophagy inhibitor, 3-methyladenine (3MA), [27] and viability in response to CDDP was evaluated. As expected (Figure 3B), inhibition of autophagy by 3MA did not modify the viability of H460 cells. Surprisingly, in H1299 cells we observed an increase in the resistance (Figure 3C). These data prompted us to consider that autophagy was a mechanism to explain the low cell death observed in H1299 cells. Next, we used a genetic approach to fully establish the role of autophagy in CDDP resistance. To this end, *Atg5* gene, which is key player in the progression of autophagy, [8] was knocked-down by using shRNA. After achieving an effective abrogation of ATG5 at the protein level in both cell lines (Figure 3D), viability was evaluated, showing almost no effect in H460 cells, while an increase in viability was observed in H1299 cells (Figures 3E and 3F) correlating with a lack of autophagy (Supplementary Figure S2B).

Therefore, this set of experiments allows us to conclude that autophagy is not mediating the observed resistant phenotype. Furthermore, it suggests that autophagy is a plausible way to explain the cell death poorly triggered by CDDP in H1299 cells.

### H1299 do not display resistance to compounds that promote autophagy

**Figure 4:**
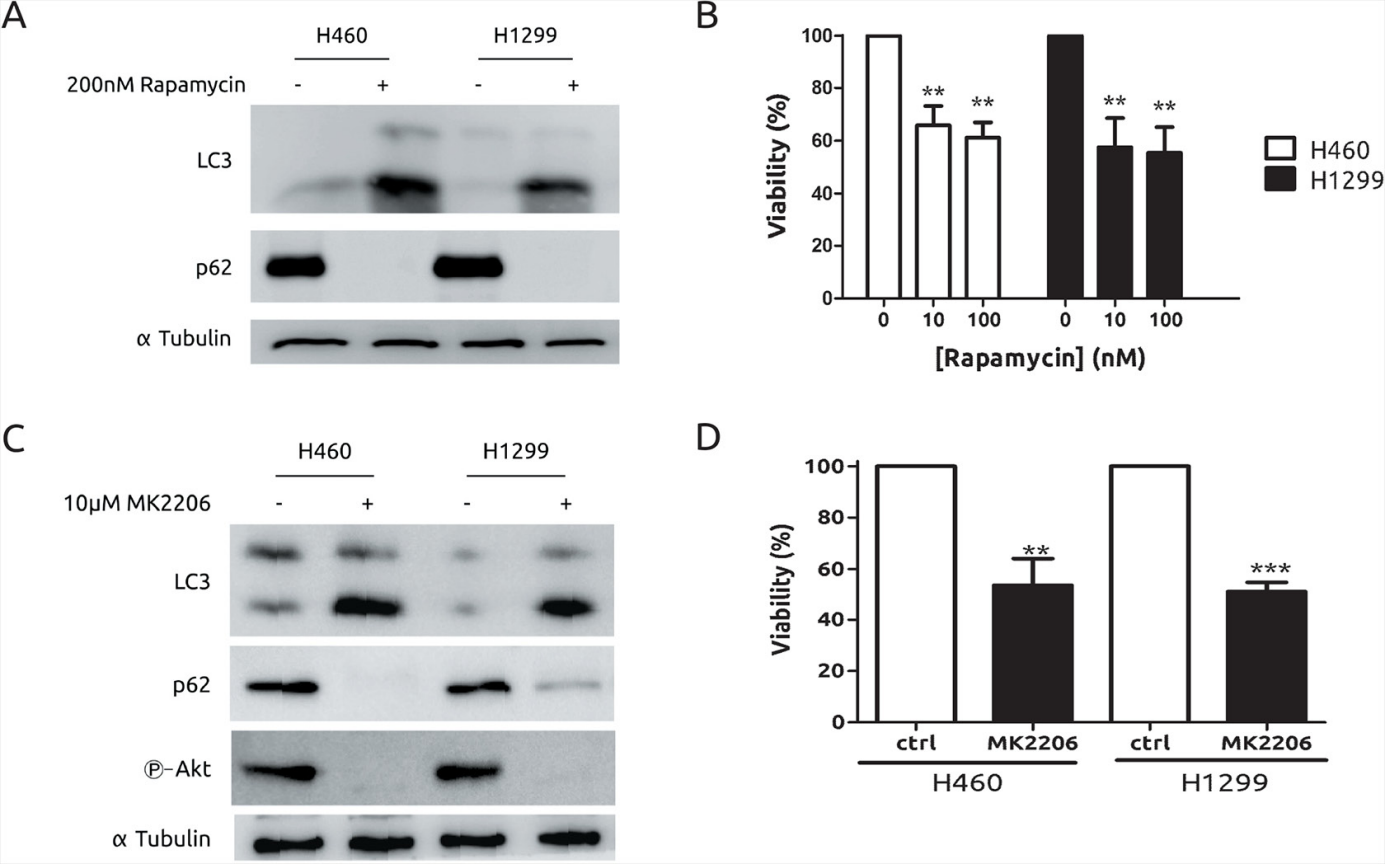
Both H460 and H1299 cells are sensitive to autophagy triggered by mTOR or Akt inhibition **A.** Cells were exposed to rapamycin for 24 h. Protein extracts were blotted with the indicated antibodies **B.** Cells were incubated with the indicated concentrations of rapamycin for 6 days. Media were replaced with fresh rapamycin every 2 days. Viability was assessed by MTT. **C.** Cells were treated with MK2206 for 24 h. Protein extracts were blotted with the indicated antibodies. **D.** Cells were treated with 10 μM MK2206 for 48 h. Viability was assessed by MTT.

**Figure 5:**
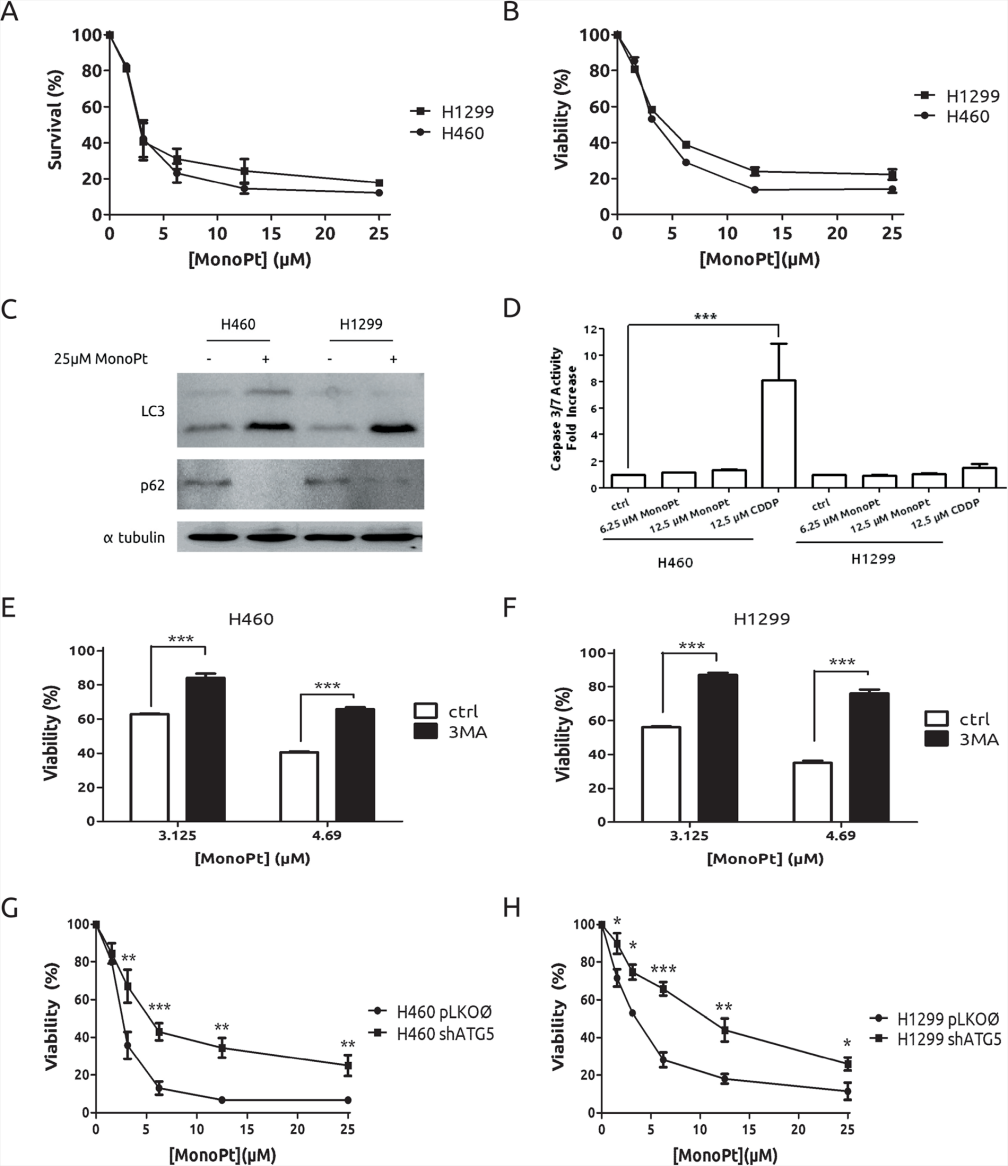
Lack of resistance to MonoPt in H1299 cells Cells were treated with indicated concentrations of MonoPt. Survival and viability were assessed by crystal violet **A.** and by MTT **B.** respectively. **C.** Cells were treated with MonoPt for 36 h and protein extracts were blotted against LC3 and p62. **D.** Cells were treated as indicated for 24 h and caspases 3 and 7 activity was evaluated. H460 **E.** and H1299 cells **F.** were co-treated with 2.5 mM 3MA at the indicated concentrations of MonoPt. Viability was assessed by MTT. **G.** ATG5-knock-down and control H460 and H1299 **H.** cells were treated with the indicated concentrations of MonoPt. Viability was measured by MTT.

In light of our previous results, we considered the possibility of exploiting autophagy as a therapeutic mechanism in our experimental model of CDDP-resistant cells. A growing body of evidences is supporting the PI3K-Akt-mTOR axis as a potent therapy target in several types of cancers including lung cancer. [28] Hence, we challenged a potent promoter of autophagy such as rapamycin. As expected, both cell lines showed a marked induction of autophagy (Figure 4A) and a similar grade of toxicity in response to rapamicyn (Figure 4B). Next, treatment with the Akt inhibitor MK2206, known to promote autophagic cell death [29] was also evaluated. As it is shown in Figure 4C and 4D, both cell lines showed the same behaviour in terms of autophagy, Akt inhibition and viability upon MK2206 treatment. Therefore, these results indicate that autophagy induction is effective in both models to a similar extent and no resistance to autophagy-prone drugs was observed in this experimental system. In light of these findings we took advantage of the availability of a novel platinum derivate, monoplatin (MonoPt) able to promote specifically autophagic cell death. [30] Then, cells were exposed to MonoPt and viability was evaluated. As it is shown, H1299 and H460 cells showed similar sensitivity to this platinum compound as judged by crystal violet method (Figure 5A) or by MTT (Figure 5B). Next, we confirmed the induction of autophagy (Figure 5C and Supplementary Figure 3A) as well as the lack of apoptosis induction (Figure 5D). In this case, blockade of autophagy promotes a resistant phenotype in both cell systems by using either 3MA (Figures 5E and 5F) as well as the interference of *Atg5* (Figures 5G and 5H), correlating with an alteration in the onset of autophagy (Supplementary Figures 3B and 3C).

In summary, our data demonstrate how autophagy can be used as a novel synthetic lethally approach to overcome natural resistance to CDDP when apoptotic response is impaired.

### MonoPt-triggered autophagy is p53- and MAPKs independent

In an attempt to fully validate our strategy, the use of an autophagy-provoking compound in CDDP-resistant cells, we challenged the role of two major determinants of CDDP resistance such as p53 and MAPKs signalling axis [31].

**Figure 6:**
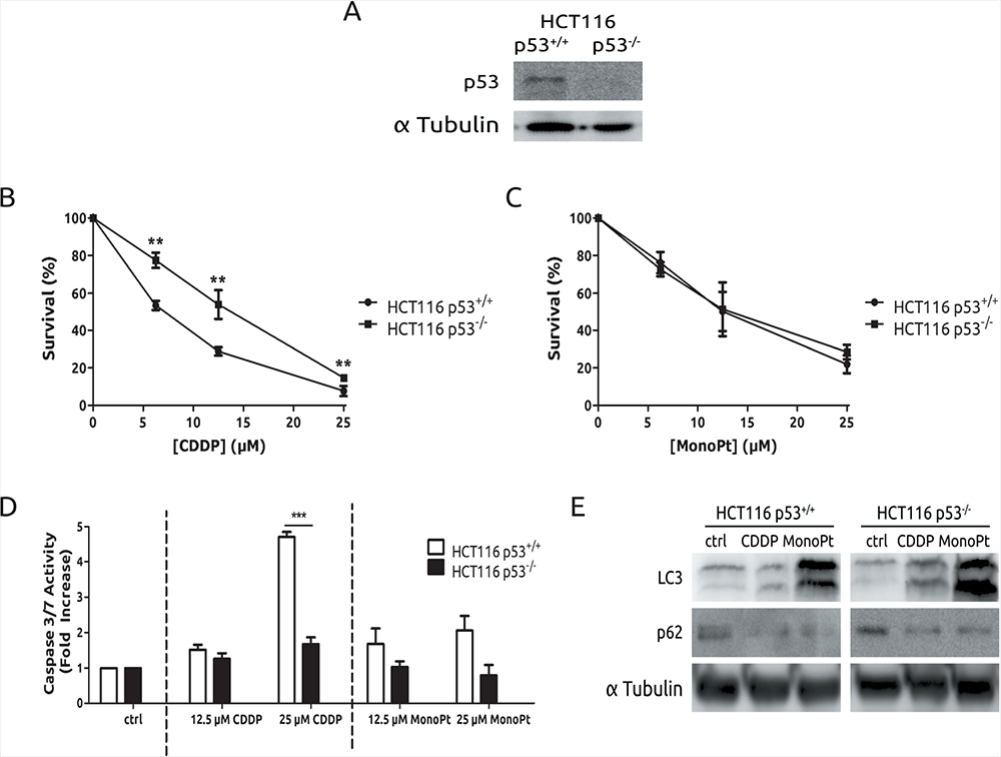
Autophagy triggered by MonoPt is p53 independent **A.** Cells were blotted to check p53 knock-out. Cells were treated at the indicated concentrations of either CDDP **B.** or MonoPt **C.** for 48 h. Survival was measured by crystal violet. **D.** Cells were treated with the indicated concentrations of either CDDP or MonoPt for 24 h. Caspase3/7 enzymatic activity was measured. **E.** Cells were treated with 25 μM of either CDDP or MonoPt for 24 h. Protein extracts were blotted with the indicated antibodies.

The role of the tumour suppressor p53 in CDDP resistance has been well established for more than 20 years. [32] In fact, in our experimental model, a strict correlation exists between lack of functional p53 and resistance. Therefore, to study the role of p53 in MonoPt-associated autophagy, we took advantage of the availability of the experimental model of HCT116 cells with both p53 alleles disrupted [33] (Figure 6A). Next, cells were exposed to CDDP or MonoPt and viability was evaluated. As expected, p53-null cells showed a clear resistance compared to p53-wt cells in response to CDDP (Figure 6B). However, in the case of MonoPt, the differences were undetectable (Figure 6C). Furthermore, apoptosis was analysed, showing a nice correlation with sensitivity to CDDP, while for MonoPt no effect was observed as judged by caspase 3/7 activity (Figure 6D). However, a marked induction of autophagy was observed in both experimental systems (Figure 6E). This set of experiments indicates that autophagy associated to MonoPt is independent of p53 activity, and could be a therapeutic alternative for patients with alterations in this tumour suppressor.

**Figure 7:**
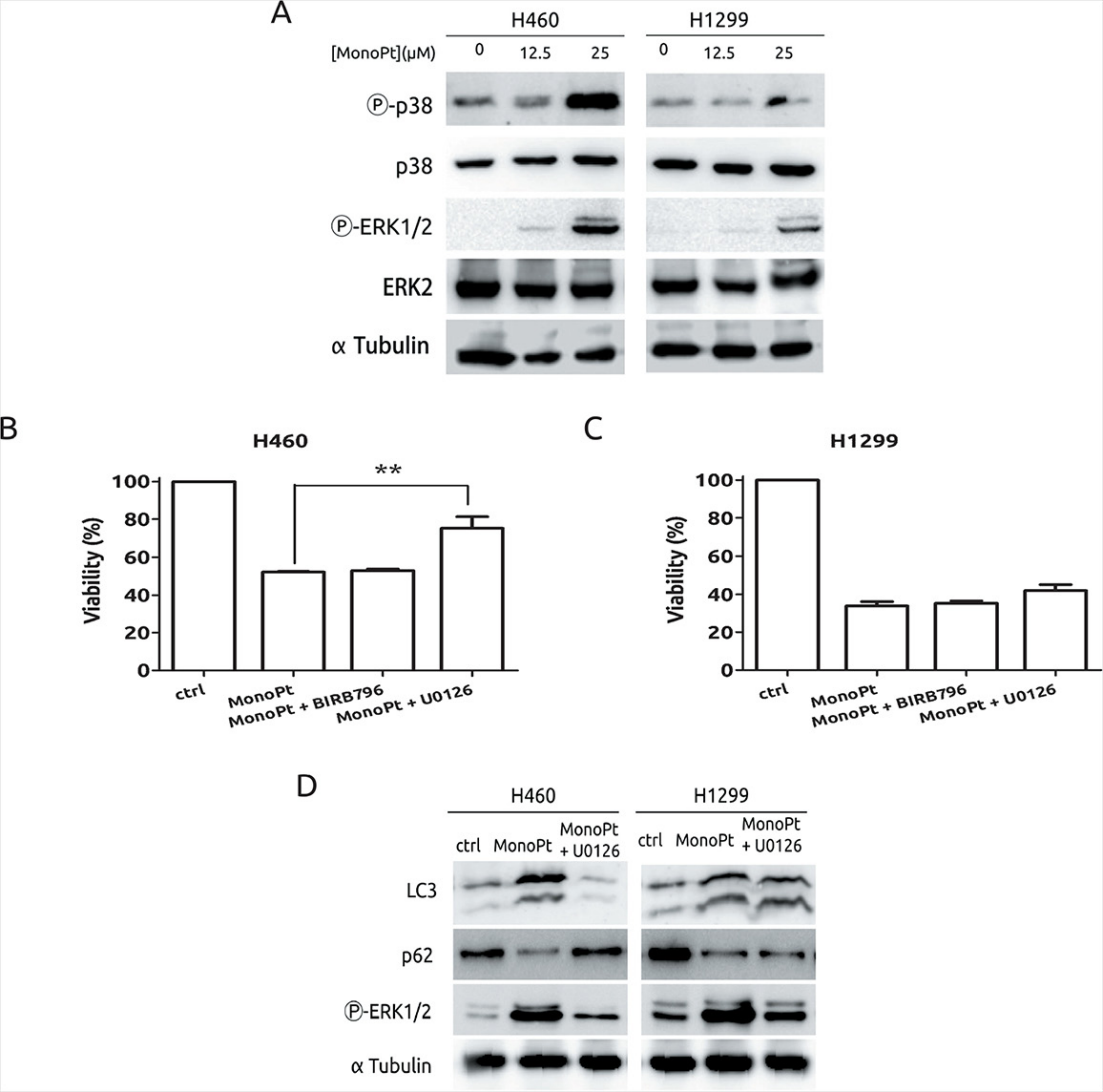
Role of MAPKs in the response to MonoPt **A.** Cells were treated with the indicated concentrations of MonoPt for 8 h. Protein extracts were blotted with the indicated antibodies. **B.** and **C.** Cells were treated with 4.69 μM MonoPt either alone or in the presence of 10 μM BIRB796 or 10 μM U0126. Viability was measured by MTT. **D.** Cells were treated with 25 μM MonoPt in the presence or absence of 10 μM U0126 for 24 hours. Protein extracts were blotted with the indicated antibodies.

Next, MAPKs were challenged in response to MonoPt. Among the several members of this family, we decided to focus onto p38 and ERK1/2, which have been shown to be implicated in the response to CDDP. [34, 35] As it is shown in Figure 7A, H460 cells displayed a marked increase in the activation of p38 MAPK in response to MonoPt, while in the case of H1299, it almost remains unaffected, with exactly the same pattern than in response to CDDP (data not shown and ref. [19]). Therefore, to fully evaluate the role of this particular MAPK we used a specific inhibitor for all p38MAPK isoforms, such as BIRB796, [36] suggesting no role for this signalling pathway in response to MonoPt (Figure 7B). Regarding to ERK1/2, both cell lines showed a marked increase in the activation of this MAPK (Figure 7A). However, inhibition of this signalling pathway by means of U0126, [37] only promotes resistance in H460, correlating with the blockade of autophagy, with no effect onto H1299 (Figures 7B, and 7D). In summary, this set of experiments demonstrates a lack of implication for p38 MAPK in the response to MonoPt and suggests that the effect of ERK1/2 seems to be cell-type specific, indicating that this signalling pathway can be excluded as universal mediator for the therapeutic effect of MonoPt.

## DISCUSSION

Several conclusions can be drawn from the present study. First, the lack of apoptosis is the main mechanism, at least in our experimental model of lung cancer, for CDDP resistance. This observation is inferred from the data obtained in H1299 cells, which were unable to trigger an apoptotic response. In this sense, promotion of apoptosis is a possible way to induce chemosensitivity in CDDP-based therapy. To this end, several approaches have been considered such as the effective abrogation of anti-apoptotic proteins, [38] the use of natural compounds, [39] modulation of specific genes [40] or the use of BH3 mimetic compounds [41, 42] among others. However, these interesting possibilities do not seem to be applicable to our current model of H1299 cells, in which, for example the use of ABT263, a BH3 mimetic, only induces a slight increase in the apoptotic response of this cell line towards CDDP with an almost undetectable shift in its sensitivity. Therefore, our data suggest that, at least in some cases (vg. H1299 cells), promotion of apoptosis is not a real therapeutic alternative.

Second, regarding to autophagy, it has been considered as a mechanism of resistance to CDDP in several pathologies including ovarian and lung cancer. [24, 43, 44] However, in addition to the data presented here, there are other evidences that do not support this idea as a general mechanism. For example, in several experimental models, this conclusion is based on a mere correlation rather than in a cause-effect demonstration (For example, refs. [45–49]). In some reports, the effect due to autophagic alterations seems to be discreet in terms of viability [43, 45, 50, 51] or the evidences presented are based only on the use of chemical inhibitors such as chloroquine or 3MA (vg. refs. [52–55]) which have been reported to also act trough an autophagy-independent mechanism even in response to CDDP. [56, 57] In addition, other evidences connecting autophagy and CDDP resistance are based onto resistant cell lines obtained by co-culturing [47, 50] or even CDDP plus other drugs. [58] In fact, in this context of acquired resistance, very different from natural resistance like in H1299 cells, previous evidences demonstrate how suppression of autophagy is a mechanism of resistance, [59] suggesting that autophagy is a potential mechanism of sensitivity. Furthermore, it is noteworthy that autophagy has been proposed as the mechanism to induce cell death in response to CDDP in the absence of key apoptotic proteins. [60] Therefore, our data demonstrate that when CDDP is unable to trigger an apoptotic response -the natural and effective mechanism of cell death associated to this drug, autophagy, -probably less effective than apoptosis in response to CDDP- is promoting cell death, indicating that both process cannot coexist. [25] Thus, our work excludes autophagy as mechanism of resistance to CDDP in our model, supporting the idea of a cytotoxic autophagy. [18, 61]

Third, our data support a lack of involvement of classical mediators of CDDP resistance (vg p53 and MAPK) in the response to MonoPt. Regarding to p53, the data obtained in H1299 as well as in HCT116 p53^−/−^, support that this approach can also be used in mutant-p53 tumours. This issue is especially important considering that alterations in p53 (mutation, inactivation, etc.) are common events in different types cancers (For more information, see http://p53.free.fr/ and http://p53.bii.a-star.edu.sg/index.php) and are also major determinants in the therapeutic response to CDDP. [32, 62, 63] Indeed, other determinants of chemo-resistance linked to p53 such as MNK2 and wee-1 [64, 65] could be overcome by MonoPt due to its p53 independent mechanism. Regarding to p38 MAPK, previous report did not detect any activation of p38 MAPK by MonoPt, [30] however our data show how p38 MAPK is activated with the same pattern than in response to CDDP (Data not shown and ref. [19]). In this regard, it is notorious that p38 MAPK, which has been proposed as a key mediator in the response to CDDP [19, 35, 66] is not likely to be extrapolated to MonoPt, probably due to the different cell death mechanism triggered. Finally, regarding to ERK1/2, previous report considered this MAPK as determinant of MonoPt toxicity [30] in the same sense as in CDDP response. [34, 43] In our experimental model, we found out that autophagy triggered by MonoPt can be either ERK1/2 dependent and independent as it is shown by the use of U0126 in both cell lines, indicating that ERK1/2 are not universal mediators of MonoPt-associated autophagy. Therefore, the definitive role of ERK1/2 in MonoPt response needs to be more deeply investigated.

**Figure 8:**
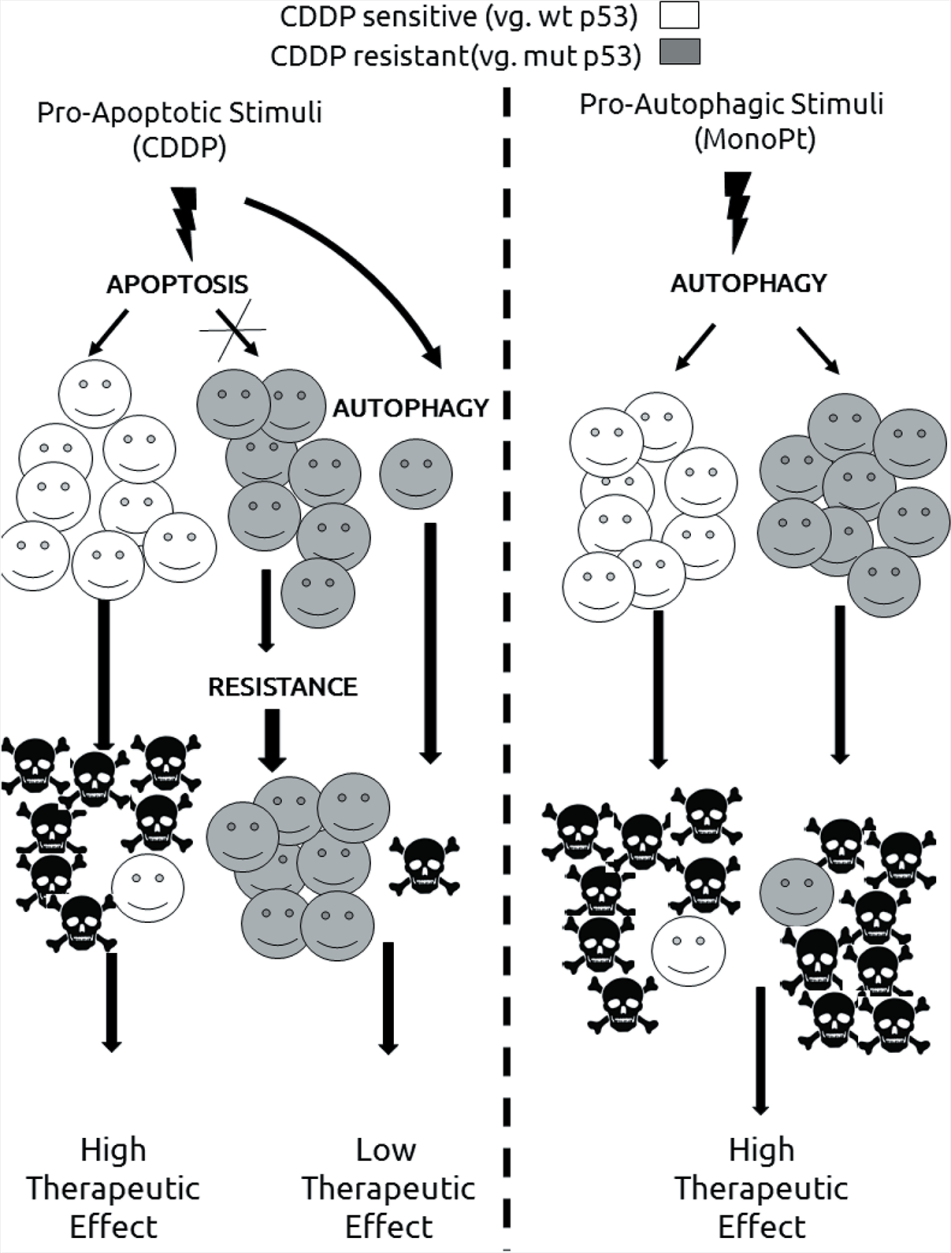
Proposed model for the use of autophagy in cisplatin-resistant tumours

Finally, a synthetic lethality approach based on the balance of autophagy/apoptosis should be considered as a novel way to overcome chemoresistance to CDDP (Figure 8). Synthetic lethality means that a combination of mutations in two genes leads to cell death, while one single mutation has no effect (For a review, see ref. [67]). This genetic principle can be extrapolated to several biological processes including cancer therapy. [68, 69] It is noteworthy, that previous works proposed synthetic lethality approaches for platinum-based therapy by using PARP-inhibitors, [70] or the use of ATR inhibitors in XRCC1 deficient cells. [71] The idea of autophagy as an agent in cancer therapy has been previously considered (For a review, see ref. [72]). But, what is more recent, is the concept of autophagy in synthetic lethality approaches for cancer therapy as in the case of renal cell carcinoma (For a review, see ref. [73]). In this sense, our report is the first evidence showing the potential role of a synthetic lethality approach based on the use of two biological processes (autophagy and apoptosis) for platinum-based therapy. This possibility is extremely interesting in tumours with a compromised apoptotic response, one of the main mechanisms of resistance to CDDP, showing a new alternative to the classical approach based on potentiation of CDDP-associated apoptosis. [74, 75]. Furthermore, considering the tumour heterogeneity known to be implicated in the therapeutic response, [76] our approach could overcome this problem as in the case of clones with different status in key molecules as p53.

In summary, we present a novel therapeutic approach to target those tumours in which the apoptotic capability of a given stimulus (CDDP) is not triggered (vg p53-mutant). In this context, the use of compounds with a high potential to trigger autophagy (vg. MonoPt) could induce cell death independently of major molecular determinants (vg p53, MAPKs). Whether our proposed mechanism could apply to other types of tumours, the putative implications in cancer therapy, as well as the role of the different components of this synthetic lethally approach, needs to be further investigated.

## MATERIALS AND METHODS

### Cell lines and cell culture

H460 and H1299 (non-small-cell lung carcinoma (NSCLC)) cells were purchased from ATCC and maintained in Dulbecco's Modified Eagle's Medium (DMEM) (D6171, Sigma Aldrich), supplemented with 10% foetal bovine serum (F7524, Sigma Aldrich), plus antibiotics (Penicillin, Streptomycin and Amphotericin B) (A5955, Sigma Aldrich) and L-Glutamine (G7513, Sigma Aldrich) at 37°C in a 5% CO_2_ atmosphere.

### Antibodies and chemicals

Antibodies against LC3 were purchased from Sigma Aldrich (L8918). Antibodies against p62/SQSTM1 (sc-28359), total p38α (sc-535), total ERK2 (sc-154), p53 (sc-126) and α-Tubulin (sc-32293) are from Santa Cruz Biotechnologies. Antibodies against phosphorylated forms of p38 MAPK (#9215) and ERK1/2 (#4377) and against ATG5 (#2630), Caspase 3 (#9662) and Caspase 9 (#9502) were purchased from Cell Signaling Technologies (CST). Antibodies against Bcl2-family members were the following ones: Bcl-2 (sc-7382, Santa Cruz), Bcl-xL (#2762, CST) and Bcl-w (sc-130701, Santa Cruz). Antobodies used against BH3-only proteins were the following: Noxa (IMG-349A, Imgenex) and Bax (sc-493, Santa Cruz).

Pan-p38 inhibitor BIRB796 (also known as Doramapimod) was purchased from Cayman Chemicals (10640). MEK1/2 inhibitor U0126 (S1102), Akt inhibitor MK2206 (S1078) and BH3 mimetic molecule ABT-263 (S1001) were from Selleckchem. Rapamycin (553210) and 3-methyladenine (3MA) (189490) were purchased from Calbiochem/Merck–Millipore and pan-caspase inhibitor Q-VD was from R&D Systems (OPH001-01M). These chemicals were diluted in DMSO and stored at −20°C (–80°C for U0126 and MK2206) until they were used.

Cis-diamminedichloroplatinum (II) (cisplatin, CDDP) was purchased from Sigma Aldrich (P4394) and diluted in bidistilled water, aliquoted and stored at −20°C until used.

Monoplatin (MonoPt) was synthesized under an inert atmosphere of dry oxygen-free nitrogen using standard Schlenk techniques. Solvents were dried from the appropriate drying agents before use, [77] and stored in presence of 4 Å molecular sieves. FAB+ mass spectrometry measurements were obtained in a Thermo MAT95XP mass spectrophotometer with magnetic sector. ^1^H NMR spectra were recorded at 298K on Varian Gemini FT-400 and Inova FT-500 spectrometers. Chemical shifts (ppm) are relative to tetramethylsilane (^1^H NMR). Coupling constants (J) are in Hertz. ^1^H−^1^H COSY spectra: standard pulse sequence with an acquisition time of 0.214s, pulse width of 10 ms, relaxation delay of 1s, 16 scans, 512 increments. In the NMR analysis, s, d, t, m, and bs denote singlet, doublet, triplet, multiplet, and broad signal, respectively. For the molar conductimetry measurements, the Λ_M_ values are given in S·cm^2^·mol^−1^ and were obtained at room temperature for 10^−3^ M solutions of the corresponding complexes in CH_3_CN, using a CRISON 522 conductimeter equipped with a CRISON 5292 platinum conductivity cell. [78] The ligand N-(tert-butoxycarbonyl)-L-methionine-N'-8-quinolylamide was prepared according to literature procedures. [79]. Although the synthesis of the MonoPt complex was described previously, [30] different attempts to follow the reported procedure in our laboratory have resulted in a mixture of products, containing a platinum complex with an unprotected -NH_2_ group due, probably, to the Boc cleavage. In order to avoid these problems, and thereby the formation of by-products, the reaction was carried out at 60°C in the presence of two equivalents of K_2_CO_3_, resulting in a new dimeric platinum complex (Di-Pt) that was obtained as pure in good yield. In a second step, the hydrolysis of Di-Pt with HCl leads to MonoPt as a pure compound. Synthesis of Di-Pt is explained in Supplementary Figure 4. [79] MonoPt was diluted in DMSO and stored at −20°C as previously described. [30]

### Survival and viability assays

An initial population of 2·10^4^ cells/well was seeded in 24-well plates. 24 hours after, media were discarded and replaced by media containing either drugs, inhibitors or both of them at the concentrations indicated in each case. After treatment (48 hours unless otherwise is indicated), survival or viability were assessed either by crystal violet method or by MTT respectively. For crystal violet, cells were washed with PBS and incubated with crystal violet reactant (C3886, Sigma Aldrich) (10 mg/ml in a distilled water-0.5% glutaraldehyde solution) for 20 min at room temperature in mild rocking. The dye was washed in running water and colorant was recovered with 10% acetic acid and transferred to transparent 96-well plates for optical density evaluation at 595 nm. MTT assays were carried out as follows: MTT reactant (Thiazolyl Blue Tetrazolium Bromide, M2128, Sigma Aldrich), at 5mg/ml in a PBS solution, was added to the cells in a 1:10 ratio (MTT solution:culture medium) and left for incubation during 1 h at 37°C. Then, media were discarded and formazan crystals adhered to the plate bottom were recovered with DMSO and transferred to a transparent 96-well plate for optical density evaluation at 570 nm.

### Apoptosis assays

For caspase activation assays, cells were plated at a density of 10^4^ cells/well in opaque 96-well plates 24 hours prior to treatment. 24 hours after treatment, activation of effector caspases 3 and 7 was evaluated with Promega's CaspaseGlo kit (G8090) following manufacturer's instructions. Resulting mixtures were quantified after 30 minutes of incubation at room temperature in a Beckton Dickinson BD 3096 luminometer.

For Annexin V/Propidium Iodide citometry assays, cells were seeded in 60-mm culture plates for a final population of 70% confluence. After treatments, cells were collected by trypsinization at the indicated times, pelleted and washed twice with ice-cold PBS. A kit form Immunostep (ANXVF-200T, BB10X-50ML and “PI”) was used for FITC-AnnexinV and Propidium Iodide staining following manufacturer's instructions. Experiments were checked for fluorescence in both green and red channels in a MACSQuant Analyzer 10 cytometer from MACS/Miltenyi Biotec. Dot plot corresponds to data obtained from a representative experiment out of three. Grouped column charts are the average of, at least, 3 independent experiments.

### Irradiation

Cells were irradiated in a Clinac Low Energy 600C linear electron accelerator from Varian by the technical staff of the University Hospital Complex of Albacete according to the indications described elsewhere. [80] For dose-response assays, 3·10^3^ cells/well were seeded in 24-well plates 24 hours prior to irradiation. Culture medium was replaced 24 hours after IR and refreshed every 2 days until the end of the experiment (6 days). Viability was evaluated by the crystal violet method.

### Western blotting

Western blot assays were performed following standard procedures. [26, 80] Briefly, cells were collected in lysis buffer (25 mM HEPES pH 7.5, 0.3 M NaCl, 1.5 mM MgCl2, 0.2 mM EDTA, 1% Triton X-100, 0.1% SDS, 0.5% deoxycholic acid, 20 mM β-glycerophosphate) plus protease and phosphatase inhibitors (2 μg/ml leupeptin, 2 μg/ml aprotinin, 1 mM PMSF and 0.1 mM Na_3_VO_4_) by using 40 μg of total cell lysates. α-Tubulin was used as a loading controls. Images show a representative experiment out of 3 with nearly identical results.

### shRNA knock-down assays, lentiviral production and infections

Plasmids codifying for short hairpin RNA (shRNA) against Atg5 were purchased from Sigma-Aldrich (SHCLNG-NM_004849). Prior to the experiments, the best performing shRNA clone was selected as judged by western blot against endogenous ATG5.

Lentiviral production and infections were as follows: HEK 293T cells were cotransfected with pSAXS (helper plasmid) and pVSV-G (envelope plasmid) lentiviral vectors along with either pLKO-puro-shATG5 or pLKO-puro empty vector (SHC001, Sigma-Aldrich) plasmids. Host cells were infected by adding packaging cells' media in the presence of 4 μg/ml polybrene from Sigma-Aldrich (H9268). 48 hours after infection cells were exposed to puromycin (ant-pr-1, Invivogene): 2 μg/ml for H460 and 3 μg/ml for H1299 cells, for at least 3 days before any assay. Infected cells were routinely maintained at the appropriate concentrations of puromycin.

### Tranfections

H1299 cells were transfected with pEX-GFP-hLC3wt or pEX-GFP-hLC3ΔG120 previously described, by using Lipofectamine LTX from Invitrogene (#15338500) following manufacturer's instructions. 48 hours later, cells were selected by using G-418 from Sigma-Aldrich (A1720) at 800 μg/ml for at least 10 days. Then, selected pools were treated and analysed as indicated in Zeiss LSM-710 confocal microscope. Images were acquired and processed using Zen 2009 Light Edition software. Images show a representative fields out of 8. The scale bars represent 25 μm.

### Data analysis

Results are represented as mean ± SEM (Standard Error of the Mean) of, at least, three independent experiments performed in triplicate. Statistical analysis was performed using the Prism 5.00 software (GraphPad) and Office Excel 2013 (Microsoft). Significance was determined using a *t*-test. The statistical significance of differences is indicated in Figures by asterisks as follows:

*⇒ *p* < 0.05; **⇒ *p* < 0.01; and ***⇒ *p* < 0.001.

## SUPPLEMENTARY FIGURES



## ABBREVIATIONS

CDDP: Cisplatin
MonoPt: Monoplatin
MAPK: Mitogen Activated Protein Kinase
DMSO: dimethyl sulfoxide
3MA: 3-Methyladenine
wt: wild type
