# Supplementary material for: Exploiting the potential of autophagy in cisplatin therapy: A new strategy to overcome resistance
Source: Oncotarget. 2015 May 6;6(17):15551–65. doi: 10.18632/oncotarget.3902 (PMC4558170; doi:10.18632/oncotarget.3902)
Supplement: Supplementary file 1 [file oncotarget-06-15551-s001.pdf]

SUPPLEMENTARY FIGURES

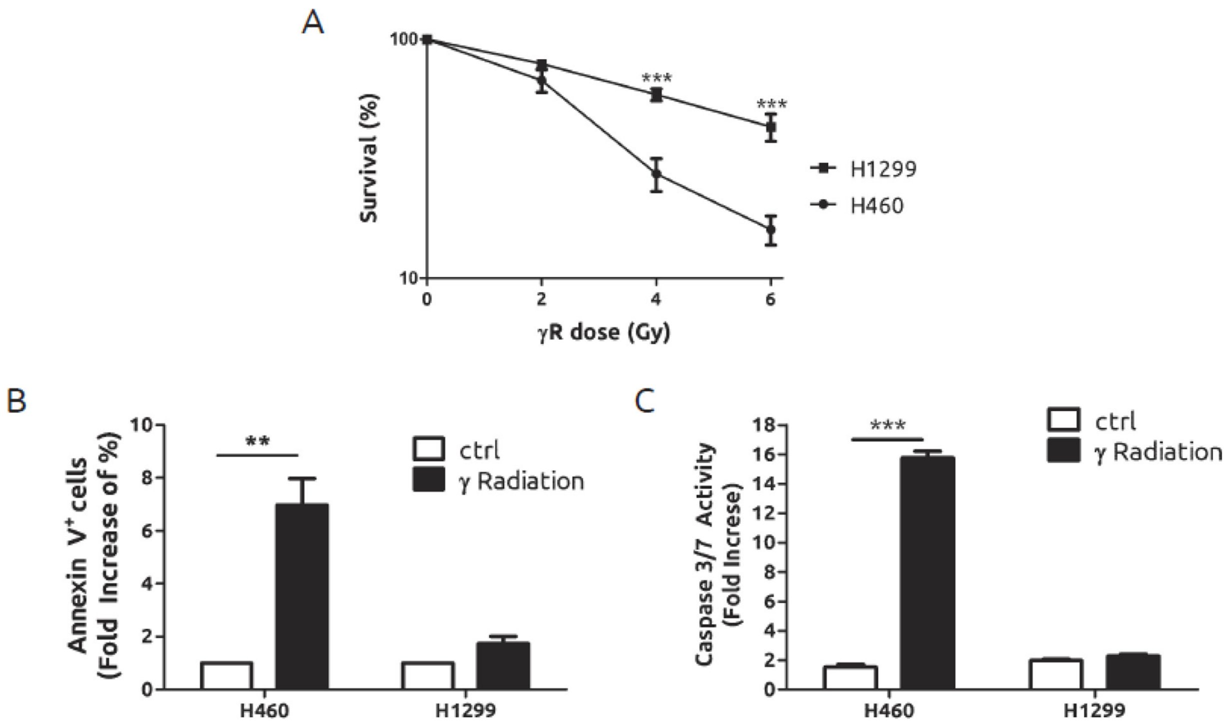

Supplementary Figure S1: A. Cells were treated with indicated doses of γ radiation and survival was measured by crystal violet 6 days after. **B.** Cells were treated with 10Gy and incubated for 36 hours before flow cytometry assay. **C.** Cells were treated as in (B) and processed for caspase activity evaluation.

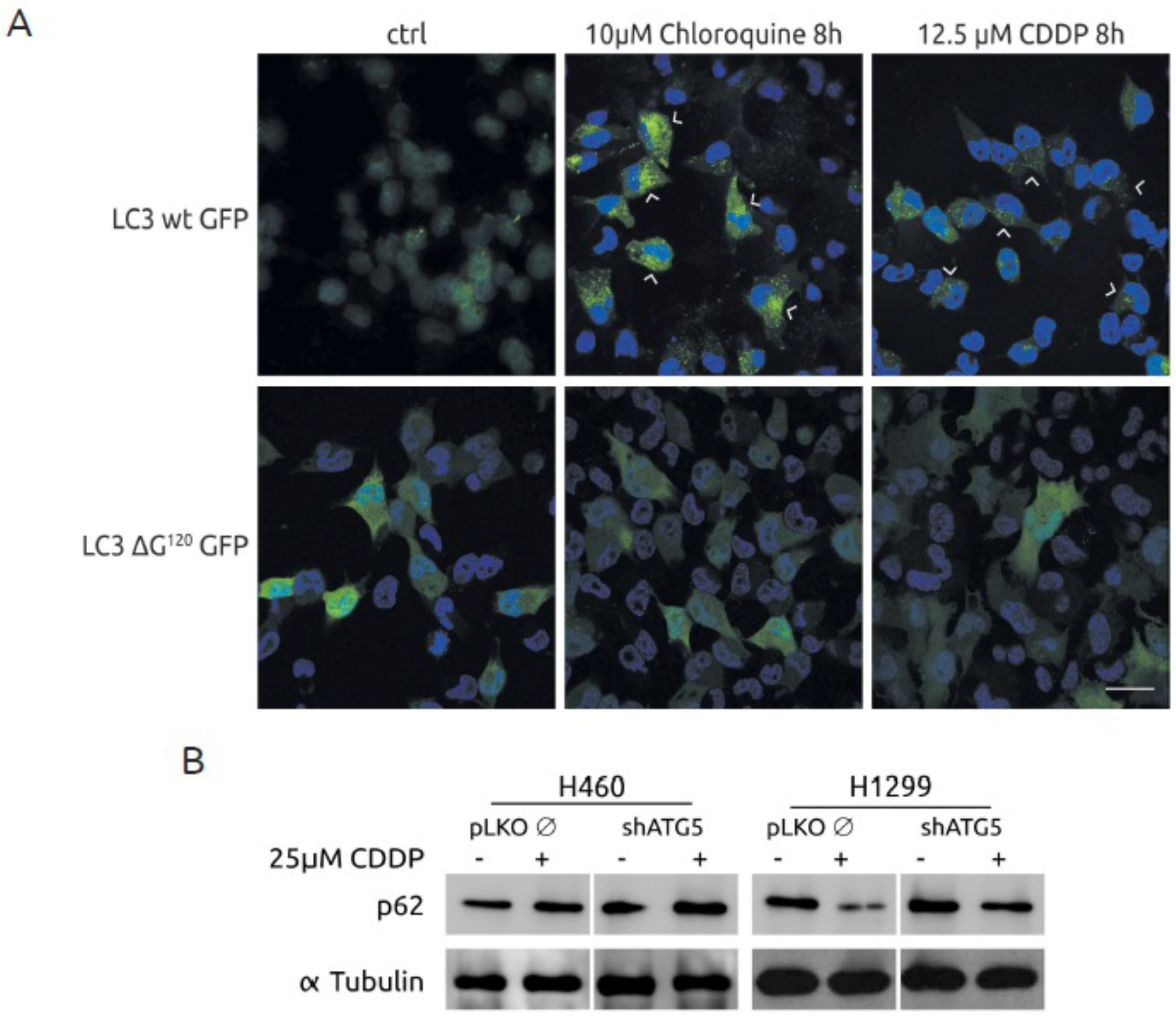

Supplementary Figure S2: A. H1299 cells were transfected with LC3wt-GFP or non-lipidable LC3ΔG120-GFP and selected with G418 for, at least, 10 days and then treated with as indicated. Arrow tips indicate puncta where LC3-GFP accumulate. **B.** Lentivirus-infected cells (empty vector or shRNA-Atg5) were treated with 25 μM CDDP for 36 h. Protein extracts were blotted with the indicated antibodies.

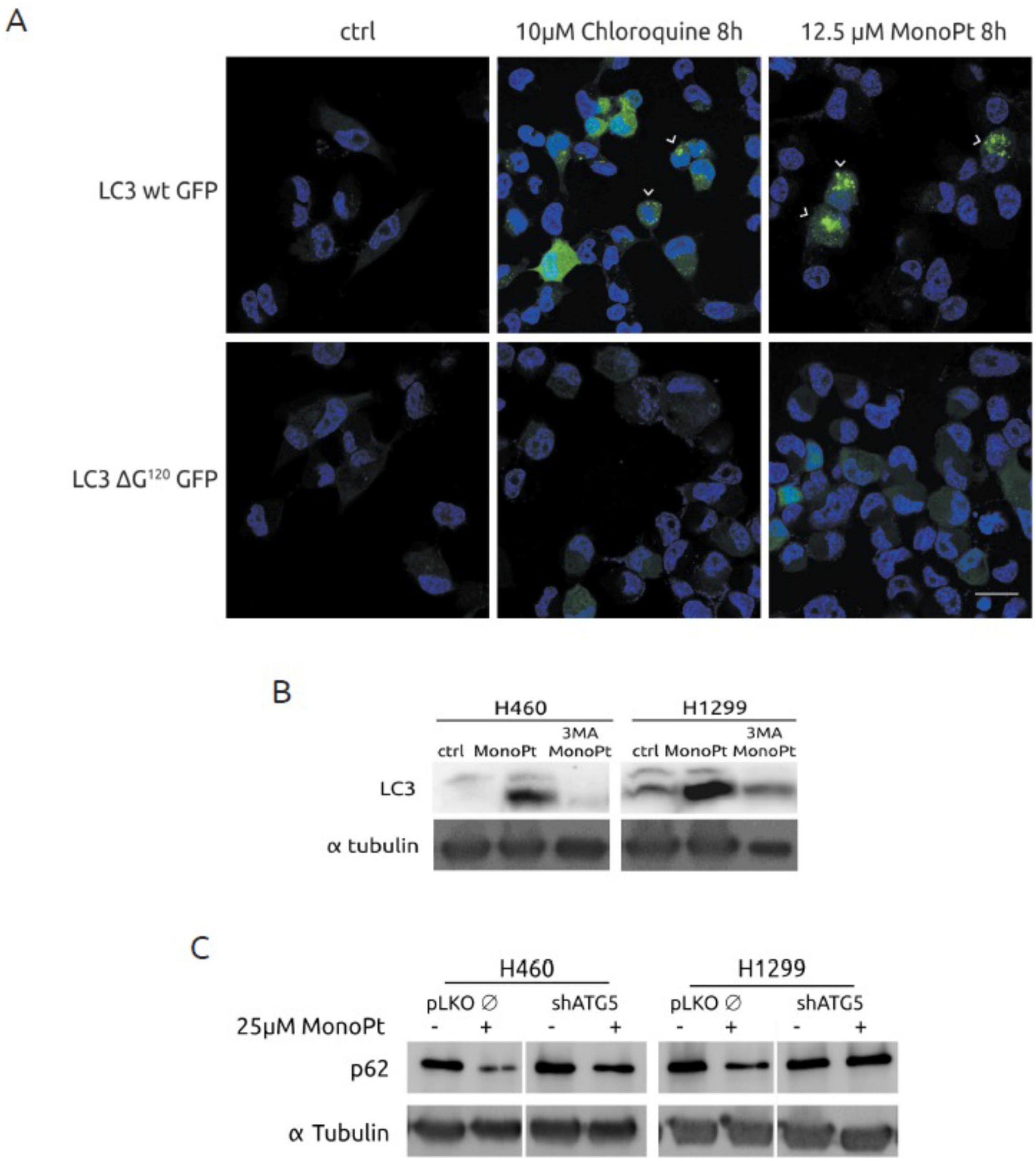

Supplementary Figure S3: A. H1299 cells were transfected with LC3wt-GFP or non-lipidable LC3ΔG120-GFP and selected with G418 for, at least, 10 days and then treated with as indicated. Arrow tips indicate puncta where LC3-GFP accumulate. **B.** Cells were treated with 25 µM MonoPt for 36 h in the presence or absence of 2.5 mM 3MA concomitantly. Protein extracts were blotted with the indicated antibodies. **C.** Lentivirus-infected cells (empty vector or shRNA-Atg5) were treated with 25 µM MonoPt for 24 hours. Protein extracts were blotted with the indicated antibodies.

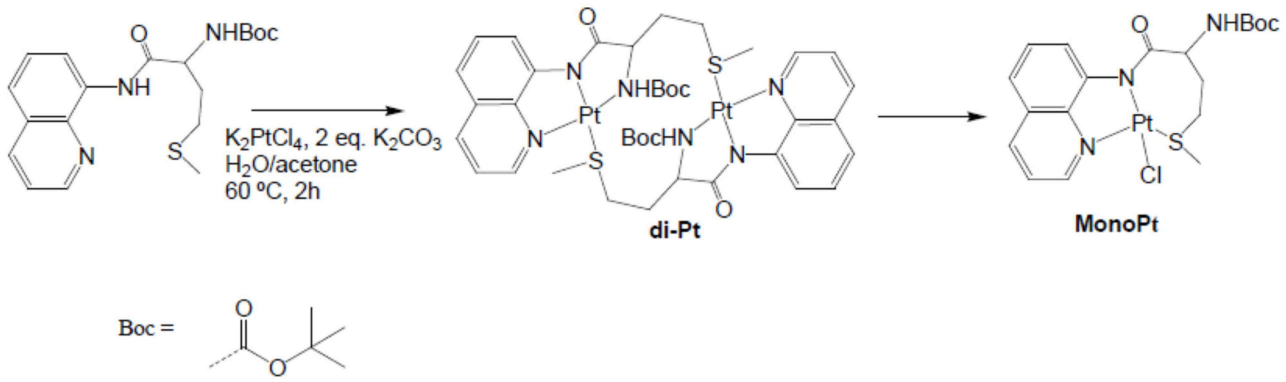

Supplementary Figure S4: The scheme depicts the synthesis of MonoPt through its intermediate form Di-Pt. Synthesis of Di-Pt. In a Schlenk tube, an aqueous solution (2 mL) of K_2PtCl_4 (83.0 mg, 0.2 mmol) and K_2CO_3 (55.3 mg, 0.4 mmol) was added to another solution of the ligand (75.1 mg, 0.2 mmol) in acetone (2 mL). The mixture was stirred at 60°C for 2 hours, when the initial intense bubbling was already finished. The yellow precipitate was filtered, washed with 5 mL of water, and dried under vacuum. The product was obtained as a yellow to orange solid. Yield: 75.0 mg, 66%. ^{1}H NMR ($CDCl_3$, 500 MHz, 298 K): 1.47 (s, 18H, Me^{tBu}), 2.60 (m, 2H, aCH2), 2.68 (bs, 2H, aCH2), 2.94(s, 6H, MeS), 3.11 (bd, J = 13.2 Hz, 2H, bCH2), 4.00 (m, 2H, bCH2), 5.27 (bs, 2H, CH), 6.37 (bs, 2H, BCH), 7.13 (d, J = 7.4 Hz, 2H, ACH), 7.51 (bt, J = 7.8 Hz, 2H, ACH), 7.77 (bd, J = 7.4 Hz, 2H, BCH), 8.15 (bs, 2H, BCH), 8.86 (d, J = 7.3 Hz, 2H, ACH) ppm. MS (FAB+, 3-NBA): m/z (assign., rel int.%): 1137 [(M+H)+, 38.7], 1037 [(M − COOtBu+2H)+, 18.7], 937 [(M − 2COOtBu+3H)+, 8.1]. Molar conductivity value, Λ_M, in CH_3CN: 22.4 $S\cdot cm^2\cdot mol^{-1}$, in accordance with a molecular complex. Synthesis of MonoPt. In a Schlenk tube, to a solution of the Di-Pt complex (70.0 mg, 0.06 mmol) in CH_2Cl_2 (5 mL), aqueous HCl (37%, 11.5 µl, 0.13 mmol) was added, changing the color of the solution from orange to bright yellow with the evolution of the reaction. After 7 hours, the solvent was evaporated under vacuum, and the resulting solid washed with diethyl ether, being the product obtained a bright yellow solid. Yield: 62.0 mg, 85%. The ^{1}H NMR spectrum is in accordance with the previous reported data.
